# Left ventricular deformation predicts major adverse cardiac events following acute myocardial infarction independently of afterload and ventricular–arterial coupling

**DOI:** 10.1007/s00392-025-02666-9

**Published:** 2025-05-26

**Authors:** Sören J. Backhaus, Jan S. Wolter, Thomas Stiermaier, Alexander Schulz, Torben Lange, Shelby Kutty, Maren Weferling, Julia M. Treiber, Johannes T. Kowallick, Gerd Hasenfuß, Andreas Rolf, Samuel Sossalla, Holger Thiele, Ingo Eitel, Andreas Schuster

**Affiliations:** 1 Department of Cardiology, Campus Kerckhoff of the Justus-Liebig-University Giessen, Kerckhoff-Clinic, Bad Nauheim, Germany; 2 German Center for Cardiovascular Research (DZHK), Partner Site Rhine-Main, Bad Nauheim, Germany; 3 Department of Cardiology and Angiology, Medical Clinic I, University Hospital Giessen, Justus-Liebig-University Giessen, Giessen, Germany; 4 University Heart Center Lübeck, Medical Clinic II (Cardiology/Angiology/Intensive Care Medicine), University Hospital Schleswig-Holstein, Lübeck, Germany; 5 German Center for Cardiovascular Research (DZHK), Partner Site Hamburg/Kiel/Lübeck, Lübeck, Germany; 6 University Medical Center Göttingen, Department of Cardiology and Pneumology, Georg-August-University Göttingen, Robert-Koch-Str. 40, 37099 Göttingen, Germany; 7 German Center for Cardiovascular Research (DZHK), Partner Site Göttingen, Göttingen, Germany; 8 Department of Medicine, Cardiovascular Division, Beth Israel Deaconess Medical Center and Harvard Medical School, Boston, USA; 9 Helen B. Taussig Heart Center, The Johns Hopkins Hospital and School of Medicine, Baltimore, MD USA; 10 FORUM Radiology, Rosdorf, Germany; 11 Department of Internal Medicine/Cardiology, Heart Center Leipzig at University of Leipzig, Leipzig, Germany; 12 FORUM Cardiology, Rosdorf, Germany

**Keywords:** Acute myocardial infarction, Afterload, Contractility, Strain, Outcome

## Abstract

**Background:**

Load dependence on left ventricular (LV) strain is under constant debate with its interference with prognostic implications remaining unclear. Consequently, we sought to investigate their interaction and prognostic value following acute myocardial infarction (AMI) using state-of-the-art cardiac magnetic resonance (CMR) imaging.

**Methods:**

In total, 1235 patients (*n* = 795 ST-elevation [STEMI] and 440 non-STEMI) underwent CMR in median 3 days following AMI. Infarct characteristics were described by CMR using tissue characterisation (infarct size, microvascular obstruction, area at risk) and deformation imaging including LV global longitudinal and circumferential strain (GLS/GCS). Non-invasive haemodynamic indices included effective arterial elastance Ea (end-systolic pressure (ESP)/stroke volume) and the non-geometric LV end-systolic afterload index NGI [(ESP × LV end-systolic volume (ESV))/LV mass] for estimation of LV afterload. LV contractility was assessed using end-systolic elastance Ees (ESP/LV ESV). Ventriculo–arterial coupling was described as Ea/Ees. Major adverse cardiac events (MACE) were recorded within the first year.

**Results:**

All haemodynamic indices were impaired in patients with MACE during follow-up compared to patients without (*p* < 0.001–0.005). Ventriculo–arterial coupling showed the highest correlation to infarct properties (infarct size *r* = 0.51, *p* < 0.001) and deformation imaging (GLS *r* = 0.54, GCS *r* = 0.72, *p* < 0.001). GLS and GCS were associated with MACE independently of all haemodynamic indices (*p* < 0.001 for all except of GCS-Ea/Ees *p* = 0.024).

**Conclusions:**

Non-invasive haemodynamic indices are associated with outcome following AMI with ventriculo–arterial coupling showing the most prominent association to infarct properties and outcome. GCS showed higher correlation to haemodynamic indices compared to GLS whilst both are independent predictors for MACE.

**Graphical abstract:**

**Figure:**
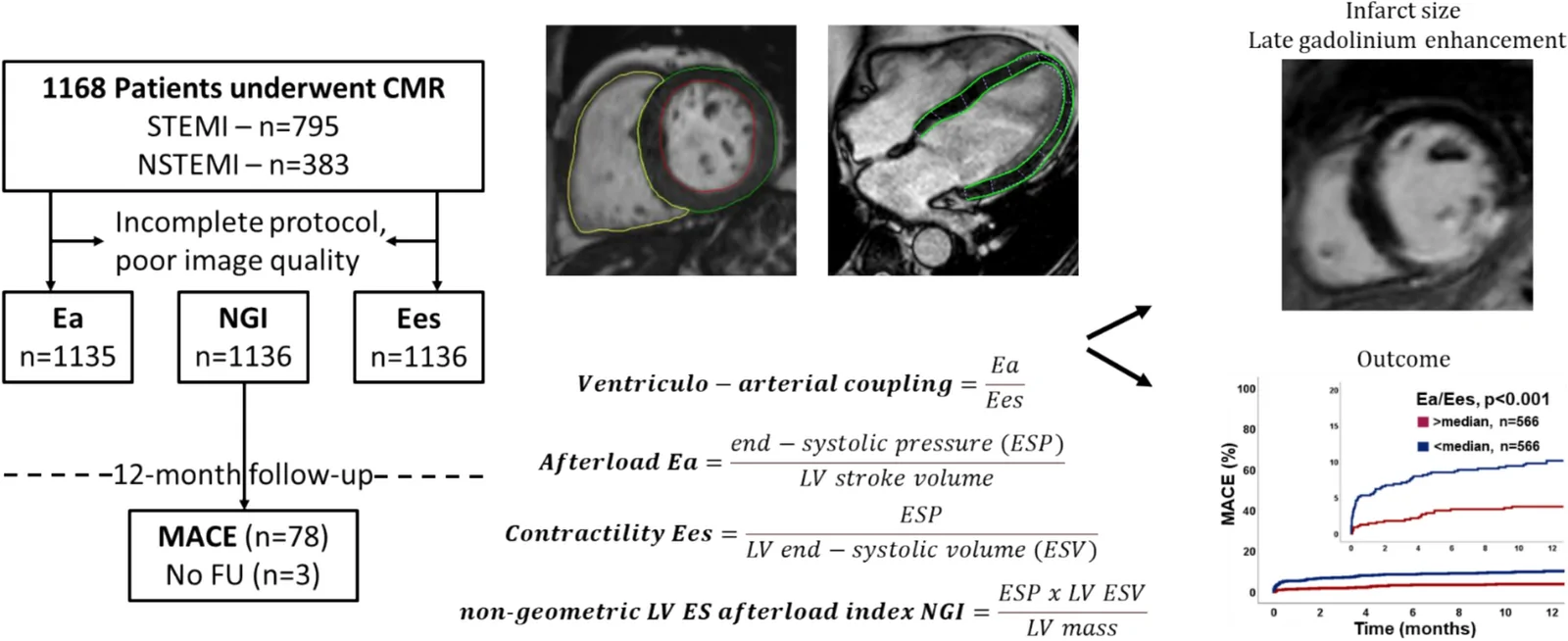

**Supplementary Information:**

The online version contains supplementary material available at https://doi.org/10.1007/s00392-025-02666-9.

## Introduction

Cardiovascular disease (CVD), of which acute myocardial infarction (AMI) is often the first clinical manifestation, is the leading cause of mortality worldwide [1]. Recurrent events following initial presentation warrant optimised risk stratification and medical therapy [2] including drug- and device-based strategies [3]. Following AMI, cardiovascular magnetic resonance (CMR) imaging allows detailed assessment of infarct properties [4] as well as deformation quantification on routinely acquired cine sequences [5] with superior prognostic power compared to left ventricular ejection fraction (LV EF) in cardiovascular disease [4, 6, 7].

Myocardial contractility on the one hand and afterload excess in adverse remodelling on the other influence outcome in heart failure [8]. Conventional afterload indices such as effective arterial elastance (Ea) take the end-systolic pressure/volume relationship into account [9]. Introduction of the non-geometric LV end-systolic afterload index (NGI) aimed for correction of differing impact of LV morphology [10]. The relationship of afterload to contractility quantifies ventriculo–arterial coupling and thus energy transfer from the ventricle into the arterial system.

Afterload dependence of right ventricular (RV) [9, 11] and LV strain [12, 13] has been demonstrated. However, data on the interaction of afterload/contractility on AMI properties and LV deformation as well as their impact on prognosis remain scarce. Consequently, we sought to evaluate their relationship to first identify the interdependence of non-invasive haemodynamic indices on infarct properties and myocardial strain as well as their interplay in major adverse cardiac event (MACE) prediction.

## Methods

### Study population

**Fig. 1:**
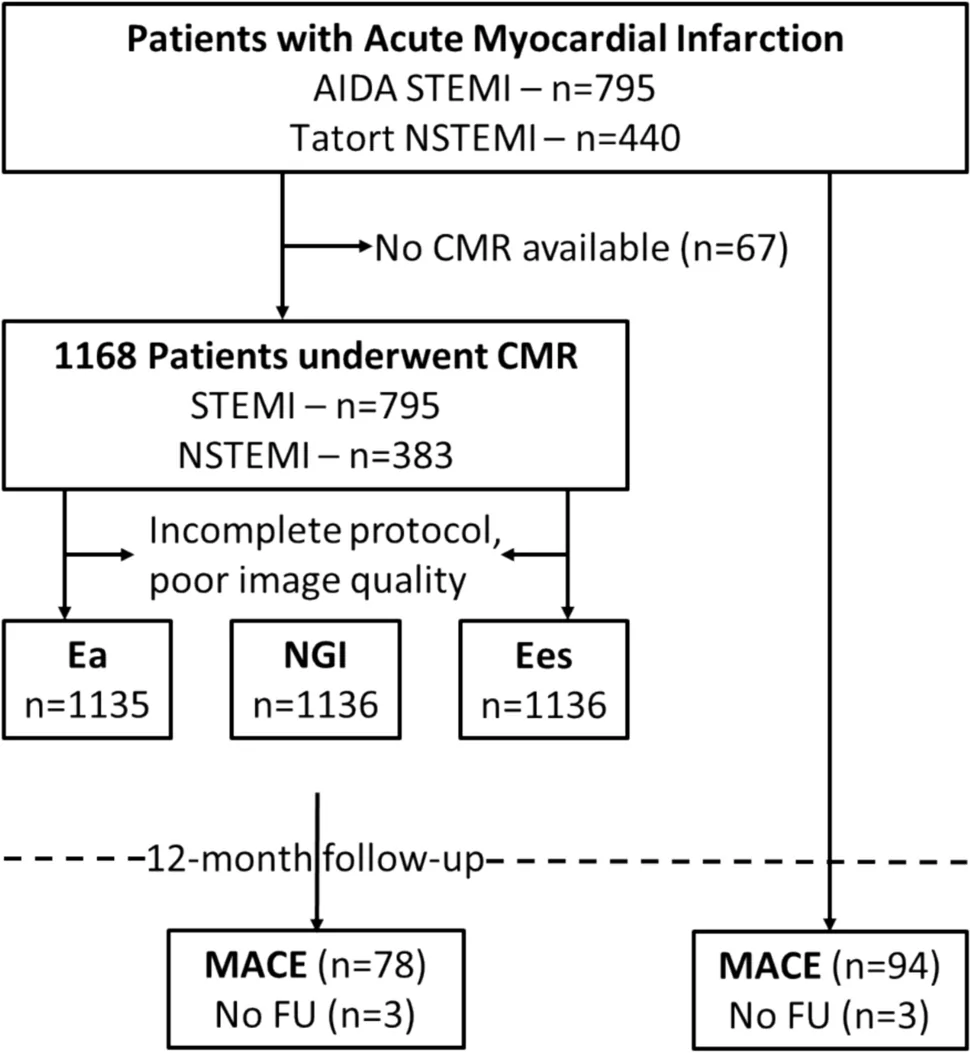
Study flow chart

This CMR substudy prospectively enrolled patients recruited to two interventional trials (AIDA STEMI [14], Abciximab Intracoronary versus intravenously Drug Application in STEMI, NCT00712101 and TATORT NSTEMI [15], Thrombus Aspiration in Thrombus Containing Culprit Lesions in Non-ST-Elevation, NCT01612312). Recruited patients additionally underwent CMR scanning following primary PCI in AMI, Fig. 1. Briefly, the AIDA STEMI trial recruited 2065 STEMI patients randomised to either intracoronary (*n* = 1032) or intravenous (*n* = 1033) abciximab application (0.25 mg/kg bodyweight) during PCI of whom 795 patients underwent additional CMR imaging after PCI. The TATORT NSTEMI trial recruited 440 NSTEMI patients randomised to aspiration thrombectomy (*n* = 221) or standard PCI (*n* = 219). All patients were recruited for CMR following PCI. Clinical endpoint was the occurrence of MACE within 12 months following the primary event. MACE included all-cause mortality, reinfarction and hospital readmission due to congestive heart failure. To avoid statistical duplication, each patient could only be attributed to one event in a hierarchical order of death > reinfarction > congestive heart failure. Events were reported by each respective study site, their classification was evaluated by a blinded committee.

The study was approved by the lead ethical committee at the University of Leipzig and all local ethical committees of involved partner sites. The study was conducted according to the principles of the Helsinki Declaration. All patients gave written informed consent before randomisation. The CMR sub-study was supported by the German Centre for Cardiovascular Research (DZHK). The data underlying the findings are available at the imaging database of the University Hospital Goettingen and access will be granted to researchers that meet the criteria for access upon formal request.

### Cardiovascular magnetic resonance imaging

CMR imaging was performed on 1.5 and 3.0 Tesla scanners within the first 10 days following PCI [15, 16]. Patients were excluded from CMR imaging according to established contraindications including creatinine clearance < 30 ml/min due to application of gadolinium-based contrast agents (GBCA) [17].

#### Morphology and volumetric analyses

Balanced steady state free precession (bSSFP) sequences were acquired for long axis (LAX) 2 and 4 chamber view (Ch) orientations as well as a short axis (SAX) stack covering both ventricles. Left ventricular mass as well as end-diastolic (EDV) and end-systolic (ESV) volumes were assessed on SAX stacks including subsequent calculation of stroke volume (SV) and ejection fraction (LVEF). 

#### Tissue characterisation

CMR sequences for tissue characterisation included T2-weighted sequences for oedema assessment (myocardium at risk) and inversion-recovery gradient echo sequences 10–20 min after the administration of GBCA for the evaluation of infarct size (IS) and microvascular obstruction (MVO) [16]. The myocardial salvage index (MSI) was calculated thereafter putting infarct size in relation to the initial myocardium at risk [18].

#### Deformation imaging

Strain analyses were performed on bSSFP sequences in an experienced and blinded core-laboratory using commercially available and extensively validated CMR-FT post-processing software (2D CPA MR, Cardiac Performance Analysis, Version 1.1.2, TomTec Imaging Systems, Unterschleissheim, Germany) [5]. LV strain analyses included global longitudinal and circumferential (GLS/GCS) strains, the former was analysed on 2/4 Ch and latter on a basal/midventricular/apical SAX slice respectively [4]. Long axis strain (LAS) assessment was performed on 2 and 4 Ch as previously described [19]. Briefly, the distance between the epicardial apical border and the middle of a line connecting the origins of the mitral leaflets was measured; LAS = ((Length endsystole − Length enddiastole)/Length enddiastole) × 100.

### Non-invasive contractility and afterload quantification

Calculations were performed based on cardiac volumes indexed to body surface area. Non-invasive quantification of afterload included calculation of effective arterial elastance [9] $\mathrm{Ea}=\frac{\text{end-systolic pressure (ESP)}}{{\rm SV}}$ and the non-geometric LV end-systolic afterload index $\mathrm{NGI}=\frac{\mathrm{ESP} \times {\text{LV ESV}}}{\text{LV mass}}$. LV contractility was estimated using end-systolic elastance [9] $\mathrm{Ees}=\frac{\mathrm{ESP}}{\text{LV ESV}}$. Ventriculo–arterial coupling was assessed using the ratio of afterload by the means of Ea to contractility Ees. ESP was derived from non-invasive blood pressure monitoring and calculated as follows: 0.98 × [DBP + (SBP − DBP)/3] + 11 [12].

### Statistical analyses

Categorical variables are reported in absolute numbers with corresponding percentage values and were compared using the Chi-squared test. Continuous variables were tested for normal distribution using the Shapiro–Wilk test, are reported in median values with interquartile range (IQR) and were compared using the Mann–Whitney *U* test. Correlations were assessed using the spearman’s rank correlation coefficient and assumed poor ≤ 0.2, fair 0.2–0.5, moderate 0.5–0.8 and strong ≥ 0.8 [20]. MACE and mortality prediction was evaluated using 1. uni- and multivariate Cox regression analyses which are reported as hazard ratio (HR) with corresponding 95% confidence interval (CI) as well as 2. area under the curve (AUC) analyses and 3. Kaplan–Meier plots with log-rank-testing. *p* values provided are two-sided and considered significant below 0.05. Calculations were performed using SPSS version 27.0 (IBM, Armonk, New York, USA) and MedCalc version 20.027 (MedCalc Software bvba, Ostend, Belgium).

## Results

### Study population

Out of 1235 initially recruited patients, 1136 CMR data sets were available for volumetric analyses, Fig. 1, which were acquired in median 3 days (IQR 2–4) following AMI. During the 12-months follow-up period, 78 MACE were noted, 3 patients were lost to follow-up. Patients with MACE during follow-up were significantly older (*p* < 0.001) with higher incidence of hypertension (*p* = 0.014) and diabetes (*p* = 0.012). On clinical assessment, Killip class on admission (*p* < 0.001) was significantly higher in patients with MACE. On angiography, the number of diseased vessels (*p* = 0.014) was higher in patients with MACE whilst TIMI flow pre or post intervention was similar in both groups (*p* = 0.539 and 0.166), Table 1 as well as supplementary Table S1–3 for baseline characteristics according to the median of Ea, NGI and Ees.

**Table 1:** Baseline characteristics

Variable	All patients *n* = 1136	MACE *n* = 78	No MACE *n* = 1055	Significance *p*
Age	64 (53, 72)	72 (61, 77)	63 (52, 72)	**< 0.001**
Sex (m)	858/1136 (75.5)	52/78 (66.7)	804/1055 (76.2)	0.058
Cardiovascular risk factors				
Active smoking	457/1054 (43.4)	21/70 (26.9)	434/981 (44.2)	**0.020**
Hypertension	807/1133 (71.2)	65/78 (83.3)	739/1052 (70.2)	**0.014**
Hyperlipoproteinemia	434/1129 (38.4)	27/78 (34.6)	405/1048 (38.6)	0.480
Diabetes	262/1133 (23.1)	27/78 (34.6)	234/1052 (22.2)	**0.012**
Body mass index (kg/m^2^)	27.5 (24.9, 30.4)	27.3 (25.3, 31.0)	27.5 (24.9, 30.3)	0.672
Previous myocardial infarction	79/1135 (7.0)	5/78 (6.4)	72/1054 (6.8)	0.887
Previous PCI	99/1136 (8.7)	5/78 (6.4)	92/1055 (8.7)	0.482
Previous CABG	22/1136 (1.9)	2/78 (2.6)	20/1055 (1.9)	0.680
Atrial fibrillation	63/1132 (5.5)	11/78 (14.1)	52/1051 (4.9)	**< 0.001**
ST-segment elevation	782/1136 (68.8)	53/78 (67.9)	729/1055 (69.1)	0.832
Systolic blood pressure (mmHg)	134 (120, 150)	130 (110, 150)	135 (120, 150)	0.093
Diastolic blood pressure (mmHg)	80 (70, 90)	77 (65, 84)	80 (70, 90)	**0.024**
Heart rate (beats/min)	76 (67, 86)	80 (70, 95)	76 (67, 86)	**< 0.001**
Time symptoms to balloon^a^ (min)	180 (109, 311)	191 (116, 370)	180 (106, 310)	0.309
Door-to-balloon time^a^ (min)	30 (22, 42)	28 (23, 40)	30 (22, 43)	0.529
Killip class on admission				**< 0.001**
1	1011/1136 (89.0)	51/78 (65.4)	957/1055 (90.7)	
2	87/1136 (7.7)	18/78 (23.1)	69/1055 (6.5)	
3	22/1136 (1.9)	5/78 (6.4)	17/1055 (1.6)	
4	16/1136 (1.4)	4/78 (5.1)	12/1055 (1.1)	
Diseased vessels				**0.014**
1	564/1136 (49.6)	29/78 (37.2)	533/1055 (50.5)	
2	343/1136 (30.2)	24/78 (30.8)	319/1055 (30.2)	
3	229/1136 (20.2)	25/78 (32.1)	203/1055 (19.2)	
Affected artery				0.197
Left anterior descending	463/1135 (40.8)	41/78 (52.6)	422/1055 (40.0)	
Left circumflex	238/1135 (21.0)	15/78 (19.2)	221/1055 (20.9)	
Left main	5/1135 (0.4)	0/78 (0.0)	5/1055 (0.5)	
Right coronary artery	422/1135 (37.1)	21/78 (26.9)	400/1055 (37.9)	
Bypass graft	8/1135 (0.7)	1/78 (1.3)	7/1055 (0.7)	
TIMI flow grade before PCI				0.539
0	573/1136 (50.4)	45/78 (57.7)	526/1055 (49.9)	
1	128/1136 (11.3)	6/78 (7.7)	122/1055 (11.6)	
2	232/1136 (20.4)	14/78 (17.9)	217/1055 (20.6)	
3	203/1136 (17.9)	13/78 (16.7)	190/1055 (18.0)	
TIMI flow grade after PCI				0.166
0	21/1136 (1.8)	1/78 (1.3)	20/1055 (1.9)	
1	23/1136 (2.0)	4/78 (5.1)	19/1055 (1.8)	
2	87/1136 (7.7)	8/78 (10.3)	79/1055 (7.5)	
3	1005/1136 (88.5)	65/78 (83.3)	937/1055 (88.8)	
Medication				
Aspirin	1135/1136 (99.9)	78/78 (100)	1054/1055 (99.9)	0.786
Clopidogrel/prasugrel/picagrelor	1135/1135 (100)	78/78 (100)	1055/1055 (100)	
Betablocker	1086/1134 (95.8)	76/78 (97.4)	1007/1053 (95.6)	0.446
ACE-inhibitor/AT-1 antagonist	1045/1134 (92.2)	74/78 (94.9)	969/1053 (92.0)	0.365
Aldosterone antagonist	150/1134 (13.2)	24/78 (30.8)	126/1053 (12.0)	**< 0.001**
Statin	1087/1134 (95.8)	75/78 (96.2)	1009/1053 (95.8)	0.887
Time to MRI (days)	3 (2, 4)	3 (2, 4)	3 (2, 4)	**0.022**

Data presented as *n*/*N* (%) or median (interquartile range). *p* values were calculated for the comparison between patients with and without MACE. Numbers in bold type indicate a significant difference

*MACE* major adverse clinical event, *CABG* coronary artery bypass graft, *PCI* percutaneous coronary intervention, *TIMI* thrombolysis in myocardial infarction

^a^Only assessed in STEMI

In terms of tissue characterisation, infarct size most prominently differed between patients with and without MACE during follow-up (20.3% vs 13.0%, *p* < 0.001) paralleled by higher AAR and lower MSI in patients with MACE, Table 2.

**Table 2:** Cardiac characterisation

Variable	All patients *n* = 1136	MACE *n* = 78	No MACE *n* = 1055	Significance *p*
CMR-derived volumetry				
EDV (ml/m^2^ BSA)	73.4 (62.0, 85.8)	75.3 (67.3, 89.2)	73.2 (61.9, 85.4)	0.114
ESV (ml/m^2^ BSA)	35.6 (27.7, 45.9)	45.0 (32.1, 53.9)	35.1 (27.6, 45.3)	**< 0.001**
SV (ml/m^2^ BSA)	36.2 (30.6, 42.3)	33.1 (25.2, 38.1)	36.5 (30.9, 42.6)	**< 0.001**
EF (%)	50.6 (43.4, 57.5)	40.6 (33.1, 52–3)	50.9 (44.2, 57.6)	**< 0.001**
Mass (g/m^2^ BSA)	66.5 (57.6, 76.3)	68.3 (58.6, 78.7)	66.4 (57.4, 76.3)	0.533
CMR-derived infarct characterisation				
IS	13.2 (5.4, 21.7)	20.3 (9.0, 28.9)	13.0 (5.3, 21.3)	**< 0.001**
MVO	0.4 (0.0, 2.0)	0.8 (0.0, 2.7)	0.3 (0.0, 1.9)	0.055
AAR	29.3 (20.3, 42.3)	33.9 (24.4, 45.7)	29.1 (20.2, 42.2)	**0.048**
MSI	54.8 (34.2, 75.4)	45.6 (23.5, 72.3)	55.5 (35.1, 75.4)	**0.028**
CMR-derived deformation				
GLS (%)	− 16.4 (− 12.4, − 20.1)	− 11.6 (− 8.3, − 17.1)	− 16.6 (− 12.9, − 16.6)	< 0.001
GCS (%)	− 23.9 (− 19.1, − 23.9)	− 18.3 (− 14.6, − 24.1)	− 24.2 (− 19.5, − 28.9)	< 0.001
LAS (%)	− 11.1 (− 8.5, − 13.2)	− 8.3 (− − 5.9, − 11.1)	− 11.2 (− 8.7, − 13.2)	< 0.001
Afterload				
Ea	2.9 (2.4, 3.5)	3.1 (2.6, 3.9)	2.9 (2.4, 3.5)	0.005
NGI	57.4 (44.9, 71.1)	65.6 (49.3, 75.7)	56.8 (44.3, 70.5)	< 0.001
Contractility				
Ees	2.9 (2.2, 3.9)	2.3 (1.8, 3.5)	3.0 (2.3, 4.0)	< 0.001
Ventriculo–arterial coupling				
Ea/Ees	1.0 (0.7, 1.3)	1.5 (0.9, 2.0)	1.0 (0.7, 1.3)	**< 0.001**

Data presented as median (interquartile range). *p* values were calculated for the comparison between patients with and without MACE using the Mann–Whitney *U* test. Numbers in bold type indicate a significant difference

*EDV/ESV* end-diastolic/systolic volume, *SV* stroke volume, *EF* ejection fraction, *BSA* body surface area, *IS* infarct size, *MVO* microvascular obstruction, *AAR* area at risk, *MSI* myocardial salvage index, *GLS/GCS* global longitudinal/circumferential strain, *Ea* effective arterial elastance, *NGI* non-geometric left ventricular end-systolic afterload index, *Ees* end-systolic elastance

### Afterload, contractility, and ventriculo–arterial coupling

#### Infarct characteristics

Correlation of afterload as assessed by Ea and infarct characteristics IS, MVO and MSI was poor but statistically significant (*r* ≤ 0.2, *p* ≤ 0.011) with higher afterload being associated to larger infarct size. Correlation increased to fair coefficients after correction for LV mass using NGI with highest correlation to IS (*r* = 0.35, *p* < 0.001).

Similarly, correlation between contractility and infarct characteristics was fair with larger infarct size being associated to reduced contractility and best correlation for IS (*r* = − 0.46, *p* < 0.001).

Ventriculo–arterial coupling as the relationship of afterload to contractility further improved correlation to infarct characteristics being overall fair with the best and moderate for Ea/Ees and IS (*r* = 0.51, *p* < 0.001). Larger infarct size resulted in an increased ratio of afterload to contractility, Table 3.

**Table 3:** Correlation to infarct characteristics

Variable	IS	MVO	AAR	MSI
Afterload				
Ea	− 0.08, * p* = 0.011	0.15, * p* < 0.001	0.01, * p* = 0.761	− 0.09, * p* = 0.008
NGI	− 0.35, * p* < 0.001	0.29, * p* < 0.001	0.22, * p* < 0.001	− 0.29, * p* < 0.001
Contractility				
Ees	− 0.46, * p* < 0.001	− 0.38, * p* < 0.001	− 0.31, * p* < 0.001	0.39, * p* < 0.001
Ventriculo–arterial coupling				
Ea/Ees	0.51, * p* < 0.001	0.44, * p* < 0.001	0.31, * p* < 0.001	− 0.45, * p* < 0.001

Data presented as correlation coefficient r and associated p values which were calculated according to spearman-rho

*Ea* effective arterial elastance, *NGI* non-geometric left ventricular end-systolic afterload index, *Ees* end-systolic elastance, *IS* infarct size, *MVO* microvascular obstruction, *AAR* area at risk, *MSI*: myocardial salvage index

#### Conventional cardiac functional parameters

An increase in afterload Ea correlated fairly with impaired cardiac function as appreciated by LV EF, GLS, GCS and LAS (*p* < 0.001). Correlation improved to moderate values for LV EF (*r* = − 0.67) and GCS (*r* = 0.48) but not GLS or LAS (*r* = 0.26 and 0.29) after consideration of LV mass using the NGI (*p* < 0.001 for all).

Correlation of contractility Ees was moderate to LVEF (*r* = 0.73) and GCS (*r* = − 0.55) and fair for GLS and LAS (*r* = − 0.33 for both).

Highest correlations were found for ventriculo–arterial coupling being overall moderate for LV GLS, LAS and GCS (*r* = 0.54/0.55/0.72, *p* < 0.001) and strong for LV EF (*r* = 0.98, *p* < 0.001), with impaired cardiac function being associated to an increased ratio of afterload to contractility, Table 4.

**Table 4:** Correlation to cardiac function

Variable	LVEF	LV GLS	LV GCS	LV LAS
Afterload				
Ea	− 0.32, * p* < 0.001	0.31, * p* < 0.001	0.23, * p* < 0.001	0.34, * p* < 0.001
NGI	− 0.67, * p* < 0.001	0.26, * p* < 0.001	0.48, * p* < 0.001	0.29, * p* < 0.001
Contractility				
Ees	0.73, * p* < 0.001	− 0.33, * p* < 0.001	− 0.55, * p* < 0.001	− 0.33, * p* < 0.001
Ventriculo–arterial coupling				
Ea/Ees	− 0.98, * p* < 0.001	0.54, * p* < 0.001	0.72, * p* < 0.001	0.55, * p* < 0.001

Data presented as correlation coefficient r and associated p values which were calculated according to spearman-rho

*Ea* effective arterial elastance, *NGI* non-geometric left ventricular end-systolic afterload index, *Ees* end-systolic elastance

#### Outcome following AMI

**Fig. 2:**
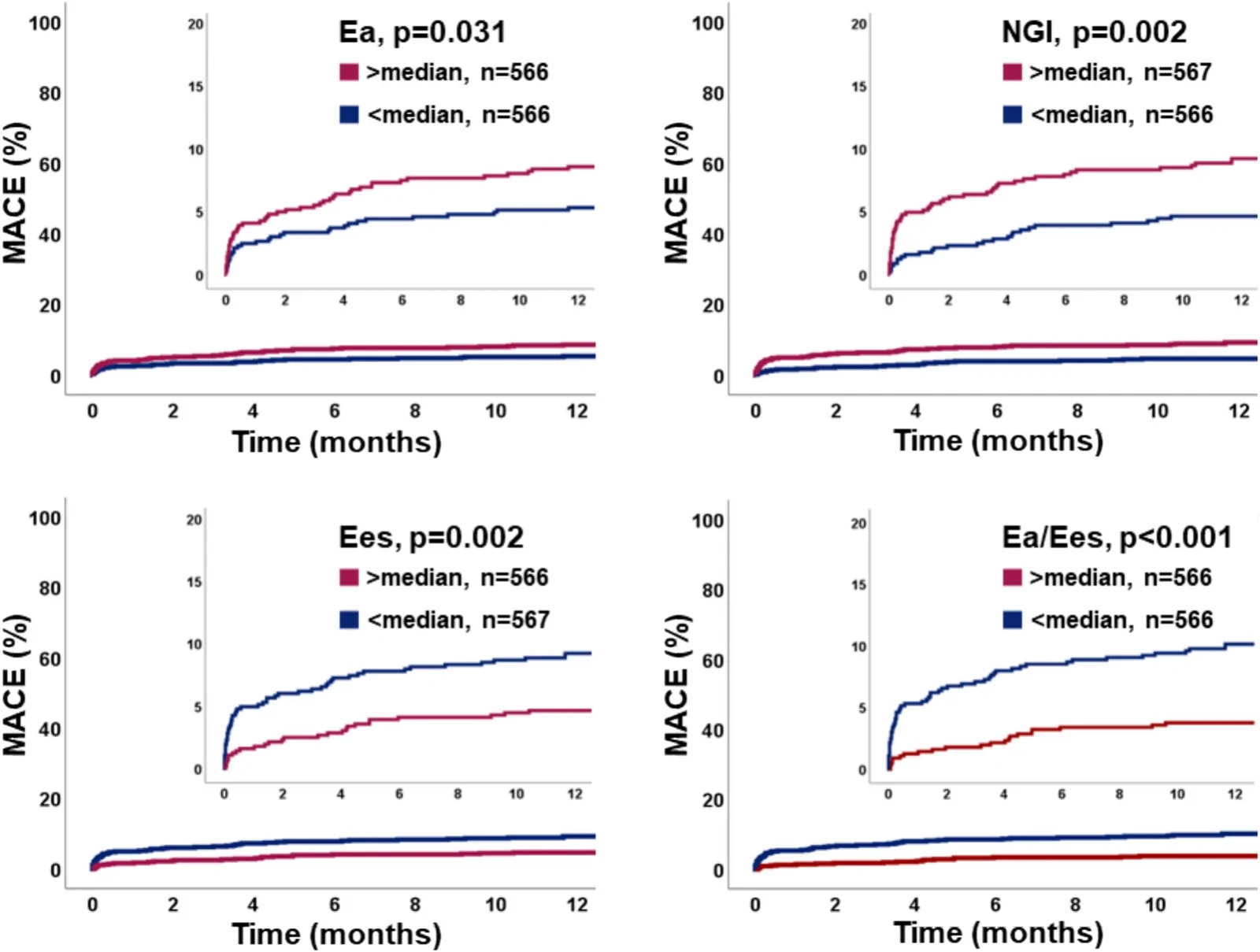
Prognostic value. The graphs show the association of afterload assessed by the means of effective arterial elastance (Ea) and the non-geometric LV end-systolic afterload index (NGI) as well as contractility assessed by end-systolic elastance (Ees) and ventriculo-arterial coupling (Ea/Ees) with major adverse cardiac events (MACE) during 12 months of follow-up following acute myocardial infarction. *p* values were calculated by log-rank testing

Following AMI, patients with MACE during follow-up showed higher afterload Ea (*p* = 0.005) and NGI (*p* < 0.001), impaired contractility Ees (*p* < 0.001) and subsequently a higher ratio of afterload to contractility in ventriculo–arterial coupling (*p* < 0.001). All non-invasive haemodynamic parameters were associated with MACE in univariate Cox-regression analyses (*p* < 0.001 for all, Table 5). Kaplan–Meier plots and corresponding log-rank testing confirmed this prognostic association, Fig. 2 with ventriculo–arterial coupling showing the most distinct impact. Ventriculo–arterial coupling outperformed Ea (AUC 0.59 vs 0.69, *p* = 0.011) as well as Ees (AUC 0.62 vs. 0.69, *p* = 0.006) regarding prognostic accuracy but not GCS (AUC 0.69 vs. 0.69, *p* = 0.756) or GLS (AUC 0.70 vs. 0.69, *p* = 0.651).

**Table 5:** Interaction of LV functional and loading parameters regarding major adverse cardiac events

Variable/univariate	Multivariate LVEF	Multivariate GLS	Multivariate GCS	Multivariate LAS
Afterload				
Ea 1.32 (1.17–1.47) *p* < 0.001	EF 0.94 (0.92–0.97) *p* < 0.001 Ea 1.10 (0.95–1.27) *p* = 0.191	GLS 1.13 (1.08–1.18) *p* < 0.001 Ea 1.16 (1.00–1.34) *p* = 0.043	GCS 1.08 (1.04–1.11) *p* < 0.001 Ea 1.19 (1.06–1.35) *p* = 0.005	LAS 1.25 (1.15–1.35) *p* < 0.001 Ea 1.17 (1.02–1.35) *p* = 0.029
NGI 1.02 (1.01–1.03) *p* < 0.001	EF 0.93 (0.91–0.96) *p* < 0.001 NGI 1.00 (0.99–1.01) *p* = 0.680	GLS 1.12 (1.08–1.17) *p* < 0.001 NGI 1.01 (1.00–1.02) *p* = 0.025	GCS 1.07 (1.04–1.11) *p* < 0.001 NGI 1.01 (1.00–1.02) *p* = 0.075	LAS 1.24 (1.14–1.34) *p* < 0.001 NGI 1.01 (1.00–1.02) *p* = 0.126
Contractility				
Ees 0.73 (0.61–0.89) *p* < 0.001	EF 0.93 (0.90–0.95) *p* < 0.001 Ees 1.17 (0.91–1.49) *p* = 0.219	GLS 1.13 (1.08–1.18) *p* < 0.001 Ees 0.87 (0.72–1.05) *p* = 0.148	GCS 1.08 (1.04–1.11) *p* < 0.001 Ees 0.86 (0.70–1.06) *p* = 0.168	LAS 1.25 (1.15–1.35) *p* < 0.001 Ees 0.90 (0.74–1.08) *p* = 0.263
Ventriculo–arterial coupling				
Ea/Ees 1.98 (1.70–2.30) *p* < 0.001	EF 0.97 (0.94–1.00) *p* = 0.062 Ea/Ees 1.56 (1.13–2.15) *p* = 0.007	GLS 1.10 (1.05–1.15) *p* < 0.001 Ea/Ees 1.62 (1.33–1.98) *p* < * p* < 0.001	GCS 1.05 (1.01–1.09) *p* = 0.024 Ea/Ees 1.71 (1.39–2.11) *p* < 0.001	LAS 1.18 (1.09–1.28) *p* < 0.001 Ea/Ees 1.62 (1.31–2.01) *p* < 0.001

Data presented as hazard ratio with 95% confidence interval and p value based on cox-regression analyses

*Ea* effective arterial elastance, *NGI* non-geometric left ventricular end-systolic afterload index, *Ees* end-systolic elastance

Univariate significant baseline characteristics and cardiovascular risk factors were integrated in a multivariate model adding LVEF, GLS (Table 6) or LAS (supplementary Table S4) and a respective loading parameter (Ea/NGI/Ees or coupling—Ea/Ees). Whilst both GLS and LAS remained independent predictors for MACE, only Ea/Ees did amongst the loading parameters. Supplementary Tables S5–10 show data on the interaction of LV functional and loading parameters according to sex, age </≥ 65 years and STEMI/NSTEMI.

**Table 6:** Clinical multivariate model for major adverse cardiac event prediction

Variable	Univariate	Multivariate Ea	Multivariate NGI	Multivariate Ees	Multivariate Ea/Ees
Age	1.05 (1.03–1.07) *p* < 0.001	1.04 (1.01–1.06) *p* = 0.004	1.04 (1.01–1.06) *p* = 0.004	1.04 (1.01–1.06) *p* = 0.004	1.04 (1.01–1.06) *p* = 0.005
Sex	1.49 (0.97–2.30) *p* = 0.067				
Smoking	0.60 (0.38–0.95) *p* = 0.029				
Hypertension	2.12 (1.22–3.68) *p* = 0.008				
HLP	0.92 (0.61–1.40) *p* = 0.702				
Diabetes	2.31 (1.53–3.49) *p* < 0.001				
Body mass index	1.03 (0.98–1.08) *p* = 0.245				
LVEF	0.94 (0.92–0.96) *p* < 0.001	0.97 (0.94–0.99) *p* = 0.009		0.96 (0.93–0.99) *p* = 0.020	
GLS	1.14 (1.09–1.18) *p* < 0.001	1.08 (1.03–1.14) *p* = 0.003	1.08 (1.03–1.14) *p* = 0.003	1.08 (1.03–1.14) *p* = 0.004	1.08 (1.03–1.14) *p* = 0.002
Respective loading parameter		0.98 (0.80–1.20) *p* = 0.843	1.00 (0.99–1.02) *p* = 0.659	1.06 (0.83–1.36) *p* = 0.622	2.25 (1.14–4.45) *p* = 0.020

The multivariate model included univariate significant parameters (univariate parameters have in parts already been published^4^) and either Ea/NGI/Ees or Ea/Ees. Data presented as hazard ratio with 95% confidence interval and *p* value based on cox-regression analyses

*Ea* effective arterial elastance, *NGI* non-geometric left ventricular end-systolic afterload index, *Ees* end-systolic elastance, *HLP* hyperlipoproteinemia, *lVEF* left ventricular ejection fraction, *GLS* global longitudinal strain

### Prognostic independence of LV strain

In multivariate analyses, LV EF, GLS, LAS and GCS were consistently and highly significantly (*p* < 0.001 for all) associated with MACE occurrence independently of non-invasive afterload and contractility haemodynamic parameters. Only GLS and LAS (*p* < 0.001 for both) as well GCS (*p* = 0.024) were predictors of MACE independent of ventriculo*–*arterial coupling, Table 5.

## Discussion

The present study investigates the interplay of non-invasive haemodynamic characterisation (afterload, contractility and ventriculo*–*arterial coupling) with cardiac deformation imaging as well as their interaction for prognostic implications following AMI.

All non-invasive haemodynamic parameters were linked to infarct properties and conventional cardiac functional parameters; in order of increasing correlation; afterload/contractility/ventriculo*–*arterial coupling. In line, the efficiency of energy transfer from the ventricular to the arterial system emerged as the best predictor for MACE following AMI amongst non-invasive haemodynamic indices. Notwithstanding, deformation imaging parameters but not LV EF were independent predictors for MACE regardless of afterload, contractility or ventriculo*–*arterial coupling.

The full spectrum of heart failure is a common entity following AMI with on the one hand diastolic preceding systolic dysfunction in the ischaemic cascade [21] and on the other hand scar tissue leading to loss of contractility. Loading conditions are an integral part in cardiac physiology. In a healthy heart, increased afterload results in increased ESV/EDV and subsequently preload which in turns increases contractility and strain by the means of the Frank Starling mechanism. However, following myocardial damage in AMI, this compensatory feature may be impaired highlighting the relationship of altered loading conditions, impaired ventriculo*–*arterial energy transfer and MACE.

Consequently, decreasing afterload is a common therapeutic target in acute and chronic heart failure [3]. Following AMI, increased afterload is commonly associated with larger infarct expansion [22] and higher degree of cardiac fibrosis [23] which may function as a substrate for ventricular arrhythmias [24] and impaired cardiac function ultimately resulting in worse prognosis. Early afterload reduction attenuates LV remodelling and is associated with improved LV functional recovery [25]. Effects of improved contractility are more prominent in non-infarcted but also—although less prominently—in infarcted segments as well [26]. Indeed, in the present population, indices for afterload and contractility were interlinked with infarct properties. Increased afterload and impaired contractility correlated to larger infarct size, MVO and reduced salvaged myocardium following PCI as well as subsequently deteriorated cardiac function. Intriguingly, using NGI instead of Ea additionally taking LV mass into consideration for afterload estimation improved correlation to tissue infarct characteristics and LV functional parameters following AMI. NGI has previously been introduced as a potentially superior parameter for non-invasive afterload quantification [8]. More accurate afterload estimation may thus explain improved correlation to infarct properties.

Not only singular afterload or contractility but also their interplay is reflected in ventriculo*–*arterial coupling to evaluate efficient energy transfer from the ventricular to the arterial system consequently allowing overall blood distribution. Indeed, ventriculo*–*arterial coupling as the ratio of Ea/Ees showed the highest association with tissue infarct characteristics. In line, the highest correlation with LV functional parameters was demonstrated for ventriculo*–*arterial coupling. Recently, a CMR-based model for non-invasive pressure–volume loops had been introduced [27] and applied in a small patient population following AMI [28]. The model demonstrated a fair correlation of ventriculo*–*arterial coupling and IS as well as MVO which was best amongst non-invasive pressure volume loops derived parameters. In line, in the present population, ventriculo*–*arterial coupling showed the highest association to tissue and functional infarct characteristics. Furthermore, ventriculo*–*arterial coupling provided incremental prognostic value for MACE prediction compared to Ea and Ees alone. In line, ventriculo*–*arterial coupling was a predictor of MACE independent of conventional cardiac functional assessments (LV EF, GLS, GCS and LAS). In the present population we could demonstrated mildly higher correlations to IS with increment in *r* = 0.10 (*r* = 0.41 vs 0.51) as well as MVO increment in *r* = 0.08 (*r* = 0.36 vs 0.44) compared to the published results based on non-invasive pressure–volume loops in a smaller AMI sample size [28]. Consequently, the present model based on LV volumetry and non-invasive blood pressure monitoring performed at least equally well compared to computed non-invasive pressure–volume loops.

GLS has a superior prognostic value across the HF spectrum compared to LVEF [6]. However, their load dependency may interfere with intrinsic prognostic power [29]. In the present population, based on over 1000 patients following AMI, we were able to demonstrate prognostic independence of GLS and GCS from afterload, contractility and ventriculo*–*arterial coupling. In contrast, LV EF was a predictor for MACE independent of afterload and contractility but not ventriculo*–*arterial coupling. This highlights the value of deformation imaging over volumetric assessments for risk assessment following AMI [4, 7, 30]. Despite its evident clinical value, GLS remains underused in clinical practise. On the one hand this may be a result from software availability, on the other hand inter-vendor agreement is critically discussed and may slow clinical implementation [31, 32]. Interestingly, LAS as a software-independent and highly reproducible approach [19] was highly comparable to GLS in terms of correlation to loading-parameters as well as independent prognostic power. This finding highlights LAS as a potential reliable surrogate parameter for GLS estimation.

### Limitations

Afterload and contractility were estimated according to surrogate parameters rather than the reference standard of LV pressure–volume loops. In line, there is no data on invasive left ventricular end-diastolic pressure. The follow-up consisted of the evaluation of MACE only, there is no data on morphological or functional changes over time from CMR.

## Conclusion

Non-invasive haemodynamic indices are associated with outcome following AMI with ventriculo*–*arterial coupling showing the most prominent association to infarct properties and outcome. Both deformation imaging and ventriculo*–*arterial coupling but not LV EF were independent predictors of MACE. First, this highlights deformation imaging as a load-independent prognostic marker. Second, it underlines the prognostic implications of both cardiac deformation as well as efficient energy transfer from the ventricle into the arterial system following AMI.

## Supplementary Information

Below is the link to the electronic supplementary material.

Supplementary file1 (DOCX 91 KB)

## Data Availability

Regarding data availability, we confirm that all relevant data are within the paper and all data underlying the findings are fully available without restriction and can be accessed at the University Medical Centre Goettingen by researchers who meet the criteria for access to confidential data.

## Abbreviations

AMI: Acute myocardial infarction
CMR: Cardiovascular magnetic resonance
CMR-FT: Cardiovascular magnetic resonance feature tracking
Ea: Effective arterial elastance
Ees: End-systolic elastance
EF: Ejection fraction
GCS: Global circumferential strain
GLS: Global longitudinal strain
IS: Infarct size
LV: Left ventricle
MACE: Major adverse cardiac event
MSI: Myocardial salvage index
MVO: Microvascular obstruction
NGI: Non-geometric LV end-systolic afterload index
RV: Right ventricle
