# Supplementary material for: Left ventricular deformation predicts major adverse cardiac events following acute myocardial infarction independently of afterload and ventricular–arterial coupling
Source: Clin Res Cardiol. 2025 May 26;115(10):1735–46. doi: 10.1007/s00392-025-02666-9 (PMC13562202; doi:10.1007/s00392-025-02666-9)
Supplement: Supplementary file 1 [file 392_2025_2666_MOESM1_ESM.docx]

| **Table S1** Baseline characteristics according to the median of afterload (Ea) | | | | |
| --- | --- | --- | --- | --- |
| **Variable** | All patients n=1136 | Ea >median | Ea <median | significance p |
| Age | 64 (53, 72) | 66 (54, 73) | 61 (51, 71) | **<0.001** |
| Sex (m) | 858/1136 (75.5) | 421/568 (74.1) | 436/567 (76.9) | 0.277 |
| **Cardiovascular risk factors** |  |  |  |  |
| Active smoking | 457/1054 (43.4) | 192/527 (36.4) | 264/526 (50.2) | **<0.001** |
| Hypertension | 807/1133 (71.2) | 448/567 (79.0) | 358/565 (63.4) | **<0.001** |
| Hyperlipoproteinemia | 434/1129 (38.4) | 237/566 (41.9) | 196/562 (34.9) | **0.016** |
| Diabetes | 262/1133 (23.1) | 159/566 (28.1) | 103/566 (18.2) | **<0.001** |
| Body mass index (kg/m²) | 27.5 (24.9, 30.4) | 27.8 (25.2, 30.8) | 26.9 (24.8, 30.1) | **0.003** |
| Previous myocardial infarction | 79/1135 (7.0) | 53/567 (9.3) | 26/567 (4.6) | **0.002** |
| Previous PCI | 99/1136 (8.7) | 60/568 (10.6) | 39/567 (6.9) | **0.028** |
| Previous CABG | 22/1136 (1.9) | 12/568 (2.1) | 10/567 (1.8) | 0.670 |
| Atrial fibrillation | 63/1132 (5.5) | 30/568 (5.3) | 33/563 (5.9) | 0.671 |
| ST-segment elevation | 782/1136 (68.8) | 363/568 (63.9) | 418/567 (73.7) | **<0.001** |
| Systolic blood pressure (mmHg) | 134 (120, 150) | 144 (130, 160) | 124 (110, 140) | **<0.001** |
| Diastolic blood pressure (mmHg) | 80 (70, 90) | 84 (78, 93) | 75 (65, 80) | **<0.001** |
| Heart rate (beats/min) | 76 (67, 86) | 80 (70, 89) | 73 (65, 83) | **<0.001** |
| Time symptoms to balloon* (min) | 180 (109, 311) | 190 (111, 344) | 165 (105, 285) | **0.044** |
| Door-to-balloon time* (min) | 30 (22, 42) | 29 (21, 42) | 30 (23, 42) | 0.456 |
| **Killip class on admission** |  |  |  | 0.238 |
| 1 | 1011/1136 (89.0) | 508/568 (89.4) | 502/567 (88.5) |  |
| 2 | 87/1136 (7.7) | 44/568 (7.7) | 43/567 (7.6) |  |
| 3 | 22/1136 (1.9) | 12/568 (2.1) | 10/567 (1.8) |  |
| 4 | 16/1136 (1.4) | 4/568 (0.7) | 12/567 (2.1) |  |
| **Diseased vessels** |  |  |  | **0.025** |
| 1 | 564/1136 (49.6) | 261/568 (46.0) | 303/567 (53.4) |  |
| 2 | 343/1136 (30.2) | 178/568 (31.3) | 164/567 (28.9) |  |
| 3 | 229/1136 (20.2) | 129/568 (22.7) | 100/567 (17.6) |  |
| **Affected artery** |  |  |  | **<0.001** |
| left anterior descending | 463/1135 (40.8) | 261/568 (46.0) | 202/567 (35.6) |  |
| left circumflex | 238/1135 (21.0) | 127/568 (22.4) | 111/567 (19.6) |  |
| left main | 5/1135 (0.4) | 4/568 (0.7) | 1/567 (0.2) |  |
| right coronary artery | 422/1135 (37.1) | 170/568 (29.9) | 251/567 (44.3) |  |
| bypass graft | 8/1135 (0.7) | 6/568 (1.1) | 2/567 (0.4) |  |
| **TIMI flow grade before PCI** |  |  |  | 0.715 |
| 0 | 573/1136 (50.4) | 278/568 (48.9) | 295/567 (52.0) |  |
| 1 | 128/1136 (11.3) | 68/568 (12.0) | 60/567 (10.6) |  |
| 2 | 232/1136 (20.4) | 120/568 (21.1) | 111/567 (19.6) |  |
| 3 | 203/1136 (17.9) | 102/568 (18.0) | 101/567 (17.8) |  |
| **TIMI flow grade after PCI** |  |  |  | 0.239 |
| 0 | 21/1136 (1.8) | 13/568 (2.3) | 8/567 (1.4) |  |
| 1 | 23/1136 (2.0) | 12/568 (2.1) | 11/567 (1.9) |  |
| 2 | 87/1136 (7.7) | 51/568 (9.0) | 36/567 (6.3) |  |
| 3 | 1005/1136 (88.5) | 492/568 (86.6) | 512/567 (90.3) |  |
| **Medication** |  |  |  |  |
| Aspirin | 1135/1136 (99.9) | 568/568 (100) | 566/567 (99.8) | 0.317 |
| Clopidogrel/Prasugrel/Ticagrelor | 1135/1135 (100) | 568/568 (100) | 567/567 (100) |  |
| Betablocker | 1086/1134 (95.8) | 543/566 (95.9) | 542/567 (95.6) | 0.773 |
| ACE-inhibitor/AT-1 antagonist | 1045/1134 (92.2) | 512/566 (90.5) | 532/567 (93.8) | **0.035** |
| Aldosterone antagonist | 150/1134 (13.2) | 93/566 (16.4) | 57/567 (10.1) | **0.002** |
| Statin | 1087/1134 (95.8) | 539/566 (95.2) | 547/567 (96.5) | 0.294 |
| Time to MRI (days) | 3 (2, 4) | 3 (2, 4) | 3 (2, 4) | **0.015** |
| Data presented as n/N (%) or median (interquartile range). P-values were calculated for the comparison between patients with and without MACE. Numbers in bold type indicate a significant difference. *only assessed in STEMI. MACE = major adverse clinical event; CABG = coronary artery bypass graft; PCI = percutaneous coronary intervention; TIMI = Thrombolysis In Myocardial Infarction | | | | |

| **Table S2** Baseline characteristics according to the median of afterload (NGI) | | | | |
| --- | --- | --- | --- | --- |
| **Variable** | All patients n=1136 | NGI >median | NGI <median | significance p |
| Age | 64 (53, 72) | 64 (52, 71) | 64 (53, 73) | 0.115 |
| Sex (m) | 858/1136 (75.5) | 429/568 (75.5) | 429/568 (75.5) | 1.000 |
| **Cardiovascular risk factors** |  |  |  |  |
| Active smoking | 457/1054 (43.4) | 238/515 (46.2) | 219/539 (40.6) | 0.068 |
| Hypertension | 807/1133 (71.2) | 405/567 (71.4) | 402/566 (71.0) | 0.881 |
| Hyperlipoproteinemia | 434/1129 (38.4) | 204/566 (36.0) | 230/563 (40.9) | 0.097 |
| Diabetes | 262/1133 (23.1) | 134/566 (23.7) | 128/567 (22.6) | 0.661 |
| Body mass index (kg/m²) | 27.5 (24.9, 30.4) | 27.4 (24.9, 30.2) | 27.6 (25.0, 30.7) | 0.529 |
| Previous myocardial infarction | 79/1135 (7.0) | 50/567 (8.8) | 29/567 (5.1) | **0.015** |
| Previous PCI | 99/1136 (8.7) | 51/568 (9.0) | 48/568 (8.5) | 0.752 |
| Previous CABG | 22/1136 (1.9) | 10/568 (1.8) | 12/568 (2.1) | 0.667 |
| Atrial fibrillation | 63/1132 (5.5) | 36/568 (6.3) | 27/564 (4.8) | 0.255 |
| ST-segment elevation | 782/1136 (68.8) | 401/568 (70.6) | 381/568 (67.1) | 0.200 |
| Systolic blood pressure (mmHg) | 134 (120, 150) | 140 (123, 152) | 130 (113, 147) | **<0.001** |
| Diastolic blood pressure (mmHg) | 80 (70, 90) | 81 (75, 90) | 78 (67, 85) | **<0.001** |
| Heart rate (beats/min) | 76 (67, 86) | 79 (70, 89) | 74 (65, 83) | **<0.001** |
| Time symptoms to balloon* (min) | 180 (109, 311) | 190 (115, 340) | 160 (100, 285) | **0.012** |
| Door-to-balloon time* (min) | 30 (22, 42) | 30 (22, 42) | 30 (21, 42) | 0.267 |
| **Killip class on admission** |  |  |  | **<0.001** |
| 1 | 1011/1136 (89.0) | 488/568 (85.9) | 523/568 (92.1) |  |
| 2 | 87/1136 (7.7) | 51/568 (9.0) | 36/568 (6.3) |  |
| 3 | 22/1136 (1.9) | 20/568 (3.5) | 2/568 (0.4) |  |
| 4 | 16/1136 (1.4) | 9/568 (1.6) | 7/568 (1.2) |  |
| **Diseased vessels** |  |  |  | 0.432 |
| 1 | 564/1136 (49.6) | 279/568 (49.1) | 285/568 (50.2) |  |
| 2 | 343/1136 (30.2) | 166/568 (29.2) | 177/568 (31.2) |  |
| 3 | 229/1136 (20.2) | 123/568 (21.7) | 106/568 (18.7) |  |
| **Affected artery** |  |  |  | **0.005** |
| left anterior descending | 463/1135 (40.8) | 262/568 (46.1) | 201/568 (35.4) |  |
| left circumflex | 238/1135 (21.0) | 110/568 (19.4) | 128/568 (22.5) |  |
| left main | 5/1135 (0.4) | 2/568 (0.4) | 3/568 (0.5) |  |
| right coronary artery | 422/1135 (37.1) | 189/568 (33.3) | 233/568 (41.0) |  |
| bypass graft | 8/1135 (0.7) | 5/568 (0.9) | 3/568 (0.5) |  |
| **TIMI flow grade before PCI** |  |  |  | **0.035** |
| 0 | 573/1136 (50.4) | 308/568 (54.2) | 265/568 (46.7) |  |
| 1 | 128/1136 (11.3) | 64/568 (11.3) | 64/568 (11.3) |  |
| 2 | 232/1136 (20.4) | 110/568 (19.4) | 122/568 (21.5) |  |
| 3 | 203/1136 (17.9) | 86/568 (15.1) | 117/568 (20.6) |  |
| **TIMI flow grade after PCI** |  |  |  | 0.235 |
| 0 | 21/1136 (1.8) | 13/568 (2.3) | 8/568 (1.4) |  |
| 1 | 23/1136 (2.0) | 12/568 (2.1) | 11/568 (1.9) |  |
| 2 | 87/1136 (7.7) | 51/568 (9.0) | 36/568 (6.3) |  |
| 3 | 1005/1136 (88.5) | 492/568 (86.6) | 513/568 (90.3) |  |
| **Medication** |  |  |  |  |
| Aspirin | 1135/1136 (99.9) | 567/568 (99.8) | 568/568 (100) | 0.317 |
| Clopidogrel/Prasugrel/Ticagrelor | 1135/1135 (100) | 568/568 (100) | 568/568 (100) |  |
| Betablocker | 1086/1134 (95.8) | 551/568 (97.0) | 535/566 (94.5) | **0.038** |
| ACE-inhibitor/AT-1 antagonist | 1045/1134 (92.2) | 526/568 (92.6) | 519/566 (91.7) | 0.569 |
| Aldosterone antagonist | 150/1134 (13.2) | 98/568 (17.3) | 52/566 (9.2) | **<0.001** |
| Statin | 1087/1134 (95.8) | 542/568 (95.4) | 545/566 (96.3) | 0.464 |
| Time to MRI (days) | 3 (2, 4) | 3 (2, 4) | 3 (2, 4) | 0.072 |
| Data presented as n/N (%) or median (interquartile range). P-values were calculated for the comparison between patients with and without MACE. Numbers in bold type indicate a significant difference. *only assessed in STEMI. MACE = major adverse clinical event; CABG = coronary artery bypass graft; PCI = percutaneous coronary intervention; TIMI = Thrombolysis In Myocardial Infarction | | | | |

| **Table S3** Baseline characteristics according to the median of contractility (Ees) | | | | |
| --- | --- | --- | --- | --- |
| **Variable** | All patients n=1136 | Ees >median | Ees <median | significance p |
| Age | 64 (53, 72) | 65 (54, 72) | 63 (52, 72) | 0.104 |
| Sex (m) | 858/1136 (75.5) | 402/568 (70.8) | 456/568 (80.3) | **<0.001** |
| **Cardiovascular risk factors** |  |  |  |  |
| Active smoking | 457/1054 (43.4) | 204/524 (38.9) | 253/530 (47.7) | **0.004** |
| Hypertension | 807/1133 (71.2) | 420/566 (74.2) | 387/567 (68.3) | **0.027** |
| Hyperlipoproteinemia | 434/1129 (38.4) | 235/564 (41.7) | 199/565 (35.2) | **0.026** |
| Diabetes | 262/1133 (23.1) | 146/566 (25.8) | 116/567 (20.4) | **0.033** |
| Body mass index (kg/m²) | 27.5 (24.9, 30.4) | 27.7 (25.0, 30.1) | 27.2 (24.8, 30.7) | 0.376 |
| Previous myocardial infarction | 79/1135 (7.0) | 29/567 (5.1) | 50/568 (8.8) | **0.015** |
| Previous PCI | 99/1136 (8.7) | 44/568 (7.7) | 55/568 (9.7) | 0.247 |
| Previous CABG | 22/1136 (1.9) | 8/568 (1.4) | 14/568 (2.5) | 0.196 |
| Atrial fibrillation | 63/1132 (5.5) | 22/566 (3.9) | 41/566 (7.2) | **0.014** |
| ST-segment elevation | 782/1136 (68.8) | 370/568 (65.1) | 412/568 (72.5) | **0.007** |
| Systolic blood pressure (mmHg) | 134 (120, 150) | 141 (127, 158) | 128 (110, 140) | **<0.001** |
| Diastolic blood pressure (mmHg) | 80 (70, 90) | 82 (75, 90) | 77 (66, 84) | **<0.001** |
| Heart rate (beats/min) | 76 (67, 86) | 76 (68, 86) | 76 (66, 86) | 0.522 |
| Time symptoms to balloon* (min) | 180 (109, 311) | 160 (102, 295) | 190 (112, 331) | 0.063 |
| Door-to-balloon time* (min) | 30 (22, 42) | 30 (21, 43) | 30 (22, 42) | 0.694 |
| **Killip class on admission** |  |  |  | **<0.001** |
| 1 | 1011/1136 (89.0) | 536/568 (94.4) | 475/568 (83.6) |  |
| 2 | 87/1136 (7.7) | 27/568 (4.8) | 60/568 (10.6) |  |
| 3 | 22/1136 (1.9) | 2/568 (0.4) | 20/568 (3.5) |  |
| 4 | 16/1136 (1.4) | 3/568 (0.5) | 13/568 (2.3) |  |
| **Diseased vessels** |  |  |  | 0.143 |
| 1 | 564/1136 (49.6) | 298/568 (52.5) | 266/568 (46.8) |  |
| 2 | 343/1136 (30.2) | 165/568 (29.0) | 178/568 (31.3) |  |
| 3 | 229/1136 (20.2) | 105/568 (18.5) | 124/568 (21.8) |  |
| **Affected artery** |  |  |  | 0.162 |
| left anterior descending | 463/1135 (40.8) | 212/568 (37.3) | 251/568 (44.2) |  |
| left circumflex | 238/1135 (21.0) | 124/568 (21.8) | 114/568 (20.1) |  |
| left main | 5/1135 (0.4) | 3/568 (0.5) | 2/568 (0.4) |  |
| right coronary artery | 422/1135 (37.1) | 226/568 (39.8) | 196/568 (34.5) |  |
| bypass graft | 8/1135 (0.7) | 3/568 (0.5) | 5/568 (0.9) |  |
| **TIMI flow grade before PCI** |  |  |  | **<0.001** |
| 0 | 573/1136 (50.4) | 241/568 (42.4) | 332/568 (58.5) |  |
| 1 | 128/1136 (11.3) | 61/568 (10.7) | 67/568 (11.8) |  |
| 2 | 232/1136 (20.4) | 136/568 (23.9) | 96/568 (16.9) |  |
| 3 | 203/1136 (17.9) | 130/568 (22.9) | 73/568 (12.9) |  |
| **TIMI flow grade after PCI** |  |  |  | 0.499 |
| 0 | 21/1136 (1.8) | 8/568 (1.4) | 13/568 (2.3) |  |
| 1 | 23/1136 (2.0) | 10/568 (1.8) | 13/568 (2.3) |  |
| 2 | 87/1136 (7.7) | 40/568 (7.0) | 47/568 (8.3) |  |
| 3 | 1005/1136 (88.5) | 510/568 (89.8) | 495/568 (87.1) |  |
| **Medication** |  |  |  |  |
| Aspirin | 1135/1136 (99.9) | 568/568 (100) | 567/568 (99.8) | 0.317 |
| Clopidogrel/Prasugrel/Ticagrelor | 1135/1135 (100) | 568/568 (100) | 568/568 (100) |  |
| Betablocker | 1086/1134 (95.8) | 536/566 (94.7) | 550/568 (96.8) | 0.075 |
| ACE-inhibitor/AT-1 antagonist | 1045/1134 (92.2) | 515/566 (91.0) | 530/568 (93.3) | 0.146 |
| Aldosterone antagonist | 150/1134 (13.2) | 43/566 (7.6) | 107/568 (18.8) | **<0.001** |
| Statin | 1087/1134 (95.8) | 547/566 (96.6) | 540/568 (95.1) | 0.184 |
| Time to MRI (days) | 3 (2, 4) | 3 (2, 4) | 3 (2, 4) | 0.352 |
| Data presented as n/N (%) or median (interquartile range). P-values were calculated for the comparison between patients with and without MACE. Numbers in bold type indicate a significant difference. *only assessed in STEMI. MACE = major adverse clinical event; CABG = coronary artery bypass graft; PCI = percutaneous coronary intervention; TIMI = Thrombolysis In Myocardial Infarction | | | | |

| **Table S4** Clinical multivariate model for major adverse cardiac event prediction | | | | | |
| --- | --- | --- | --- | --- | --- |
| **Variable** | Univariate | Multivariate  Ea | Multivariate  NGI | Multivariate  Ees | Multivariate  Ea/Ees |
| Age | 1.05 (1.03-1.07)  p<0.001 | 1.03 (1.01-1.05)  p=0.018 | 1.03 (1.01-1.06)  p=0.017 | 1.03 (1.01-1.05)  p=0.018 | 1.03 (1.00-1.05)  p=0.021 |
| Sex | 1.49 (0.97-2.30)  p=0.067 |  |  |  |  |
| Smoking | 0.60 (0.38-0.95)  p=0.029 |  |  |  |  |
| Hypertension | 2.12 (1.22-3.68)  p=0.008 |  |  |  |  |
| HLP | 0.92 (0.61-1.40)  p=0.702 |  |  |  |  |
| Diabetes | 2.31 (1.53-3.49)  p<0.001 |  |  |  |  |
| Body mass index | 1.03 (0.98-1.08)  p=0.245 |  |  |  |  |
| LVEF | 0.94 (0.92-0.96)  p<0.001 | 0.97 (0.94-1.00)  p=0.026 | 0.97 (0.94-1.00)  p=0.046 | 0.96 (0.93-0.99)  p=0.021 |  |
| LAS | 1.27 (1.18-1.37)  p<0.001 | 1.15 (1.04-1.27)  p=0.007 | 1.15 (1.04-1.27)  p=0.006 | 1.14 (1.04-1.26)  p=0.008 | 1.14 (1.04-1.26)  p=0.008 |
| Respective  loading parameter |  | 1.03 (0.85-1.26)  p=0.768 | 1.00 (0.99-1.01)  p=0.986 | 1.09 (0.85-1.40)  p=0.482 | 2.18 (1.13-4.20)  p=0.020 |
| The multivariate model included univariate significant parameters (univariate parameters have in parts already been published ^4^) and either Ea/NGI/Ees or Ea/Ees. Data presented as hazard ratio with 95% confidence interval and p-value based on cox-regression analyses. Ea: effective arterial elastance, NGI: non-geometric left ventricular end-systolic afterload index, Ees: end-systolic elastance, HLP: Hyperlipoproteinemia, LVEF: left ventricular ejection fraction, LAS: long axis strain. | | | | | |

| **Table S5** Interaction of LV functional and loading parameters regarding major adverse cardiac events in men | | | | |
| --- | --- | --- | --- | --- |
| **Variable/Univariate** | Multivariate  LVEF | Multivariate  GLS | Multivariate  GCS | Multivariate  LAS |
| **Afterload** | | | | |
| Ea  1.46 (1.17-1.82),  p<0.001 | EF 0.94 (0.91-0.97),  p<0.001  Ea 1.12 (0.88-1.44),  p=0.359 | GLS 1.12 (1.06-1.19),  p<0.001  Ea 1.18 (0.92-1.52),  p=0.200 | GCS 1.07 (1.02-1.11),  p=0.003  Ea 1.36 (1.07-1.74),  p=0.013 | LAS 1.27 (1.15-1.41),  p<0.001  Ea 1.20 (0.94-1.53),  p=0.149 |
| NGI  1.02 (1.01-1.03),  p<0.001 | EF 0.93 (0.90-0.96),  p<0.001  NGI 1.00 (0.98-1.01),  p=0.867 | GLS 1.12 (1.06-1.18),  p<0.001  NGI 1.01 (1.00-1.03),  p=0.020 | GCS 1.06 (1.01-1.11),  p=0.017  NGI 1.01 (1.00-1.03),  p=0.047 | LAS 1.26 (1.14-1.40),  p<0.001  NGI 1.01 (1.00-1.02),  p=0.135 |
| **Contractility** | | | | |
| Ees  0.73 (0.57-0.93),  p=0.012 | EF 0.92 (0.89-0.95),  p<0.001  Ees 1.23 (0.92-1.66),  p=0.169 | GLS 1.12 (1.07-1.19),  p<0.001  Ees 0.86 (0.68-1.11),  p=0.245 | GCS 1.06 (1.02-1.11),  p=0.007  Ees 0.83 (0.62-1.11),  p=0.199 | LAS 1.29 (1.16-1.42),  p<0.001  Ees 0.93 (0.73-1.18),  p=0.544 |
| **Ventriculo-arterial coupling** | | | | |
| Ea/Ees  2.63 (2.01-3.44),  p<0.001 | EF 1.00 (0.94-1.06),  p=0.892  Ea/Ees 2.50 (1.19-5.26),  p=0.015 | GLS 1.08 (1.02-1.15),  p=0.010  Ea/Ees 2.02 (1.43-2.84),  p<0.001 | GCS 1.01 (0.95-1.07),  p=0.763  Ea/Ees 2.63 (1.82-3.80),  p<0.001 | LAS 1.17 (1.04-1.31),  p=0.008  Ea/Ees 1.84 (1.26-2.70),  p=0.002 |
| Data is reported for male patients (n=856, MACE n=52) and presented as hazard ratio with 95% confidence interval and p-value based on cox-regression analyses. Ea: effective arterial elastance, NGI: non-geometric left ventricular end-systolic afterload index, Ees: end-systolic elastance. | | | | |

| **Table S6** Interaction of LV functional and loading parameters regarding major adverse cardiac events in women | | | | |
| --- | --- | --- | --- | --- |
| **Variable/Univariate** | Multivariate  LVEF | Multivariate  GLS | Multivariate  GCS | Multivariate  LAS |
| **Afterload** | | | | |
| Ea  1.23 (1.05-1.44),  p=0.010 | EF 0.95 (0.92-0.99),  p=0.006  Ea 1.07 (0.89-1.28),  p=0.500 | GLS 1.14 (1.07-1.22),  p<0.001  Ea 1.10 (0.92-1.31),  p=0.291 | GCS 1.09 (1.04-1.14),  p<0.001  Ea 1.10 (0.93-1.29),  p=0.280 | LAS 1.19 (1.05-1.35),  p=0.006  Ea 1.14 (0.95-1.36),  p=0.168 |
| NGI  1.01 (1.00-1.03),  p=0.084 | EF 0.94 (0.90-0.98),  p=0.002  NGI 0.99 (0.98-1.01),  p=0.537 | GLS 1.15 (1.07-1.23),  p<0.001  NGI 1.00 (0.99-1.02),  p=0.602 | GCS 1.09 (1.04-1.15),  p<0.001  NGI 1.00 (0.99-1.02),  p=0.711 | LAS 1.20 (1.05-1.36),  p=0.006  NGI 1.00 (0.99-1.02),  p=0.615 |
| **Contractility** | | | | |
| Ees  0.72 (0.54-0.95),  p=0.022 | EF 0.95 (0.90-0.99),  p=0.020  Ees 0.99 (0.67-1.48),  p=0.973 | GLS 1.14 (1.06-1.22),  p<0.001  Ees 0.87 (0.66-1.13),  p=0.299 | GCS 1.09 (1.04-1.14),  p<0.001  Ees 0.86 (0.64-1.15),  p=0.311 | LAS 1.18 (1.04-1.34),  p=0.011  Ees 0.83 (0.62-1.11),  p=0.198 |
| **Ventriculo-arterial coupling** | | | | |
| Ea/Ees  1.67 (1.33-2.10),  p<0.001 | EF 0.97 (0.92-1.02),  p=0.184  Ea/Ees 1.32 (0.84-2.08),  p=0.229 | GLS 1.13 (1.05-1.21),  p=0.001  Ea/Ees 1.34 (1.00-1.80),  p=0.054 | GCS 1.08 (1.02-1.14),  p=0.007  Ea/Ees 1.32 (0.98-1.77),  p=0.072 | LAS 1.15 (1.01-1.31),  p=0.032  Ea/Ees 1.47 (1.11-1.95),  p=0.007 |
| Data is reported for female patients (n=277, MACE n=26) and presented as hazard ratio with 95% confidence interval and p-value based on cox-regression analyses. Ea: effective arterial elastance, NGI: non-geometric left ventricular end-systolic afterload index, Ees: end-systolic elastance. | | | | |

| **Table S7** Interaction of LV functional and loading parameters regarding major adverse cardiac events  in patients <65 years of age | | | | |
| --- | --- | --- | --- | --- |
| **Variable/Univariate** | Multivariate  LVEF | Multivariate  GLS | Multivariate  GCS | Multivariate  LAS |
| **Afterload** | | | | |
| Ea  1.53 (1.12-2.09),  p=0.008 | EF 0.94 (0.91-0.99),  p=0.008  Ea 1.24 (0.88-1.74),  p=0.223 | GLS 1.14 (1.05-1.23),  p=0.003  Ea 1.11 (0.74-1.65),  p=0.616 | GCS 1.03 (0.96-1.09),  p=0.446  Ea 1.47 (1.06-2.04),  p=0.021 | LAS 1.27 (1.09-1.47),  p=0.002  Ea 1.24 (0.88-1.74),  p=0.214 |
| NGI  1.02 (1.00-1.04),  p=0.041 | EF 0.93 (0.89-0.98),  p=0.006  NGI 1.00 (0.97-1.02),  p=0.827 | GLS 1.13 (1.04-1.23),  p<0.001  NGI 1.01 (0.99-1.03),  p=0.408 | GCS 1.03 (0.97-1.10),  p=0.351  NGI 1.01 (0.98-1.03),  p=0.565 | LAS 1.28 (1.10-1.48),  p=0.001  NGI 1.01 (0.99-1.03),  p=0.501 |
| **Contractility** | | | | |
| Ees  0.79 (0.55-1.14),  p=0.207 | EF 0.91 (0.86-0.96),  p<0.001  Ees 1.39 (0.91-2.12),  p=0.132 | GLS 1.14 (1.05-1.24),  p=0.002  Ees 0.94 (0.66-1.35),  p=0.753 | GCS 1.03 (0.97-1.10),  p=0.364  Ees 0.90 (0.61-1.33),  p=0.584 | LAS 1.29 (1.12-1.50),  p<0.001  Ees 0.97 (0.69-1.36),  p=0.866 |
| **Ventriculo-arterial coupling** | | | | |
| Ea/Ees  2.77 (1.80-4.25),  p<0.001 | EF 1.01 (0.93-1.10),  p=0.850  Ea/Ees 3.05 (1.02-9.18),  p=0.047 | GLS 1.10 (1.01-1.21),  p=0.034  Ea/Ees 1.83 (1.01-3.33),  p=0.048 | GCS 0.96 (0.88-1.04),  p=0.309  Ea/Ees 3.35 (1.78-6.31),  p<0.001 | LAS 1.19 (1.01-1.40),  p=0.034  Ea/Ees 1.94 (1.11-3.41),  p=0.020 |
| Data is reported for patients <65 years of age (n=586, MACE n=23) and presented as hazard ratio with 95% confidence interval and p-value based on cox-regression analyses. Ea: effective arterial elastance, NGI: non-geometric left ventricular end-systolic afterload index, Ees: end-systolic elastance. | | | | |

| **Table S8** Interaction of LV functional and loading parameters regarding major adverse cardiac events  in patients ≥65 years of age | | | | |
| --- | --- | --- | --- | --- |
| **Variable/Univariate** | Multivariate  LVEF | Multivariate  GLS | Multivariate  GCS | Multivariate  LAS |
| **Afterload** | | | | |
| Ea  1.24 (1.08-1.43),  p=0.003 | EF 0.95 (0.92-0.97),  p<0.001  Ea 1.03 (0.87-1.23),  p=0.714 | GLS 1.12 (1.07-1.17),  p<0.001  Ea 1.12 (0.96-1.32),  p=0.155 | GCS 1.10 (1.06-1.14),  p<0.001  Ea 1.09 (0.93-1.27),  p=0.296 | LAS 1.21 (1.11-1.33),  p<0.001  Ea 1.14 (0.96-1.34),  p=0.131 |
| NGI  1.02 (1.01-1.03),  p=0.001 | EF 0.94 (0.91-0.97),  p<0.001  NGI 1.00 (0.98-1.01),  p=0.581 | GLS 1.12 (1.07-1.17),  p<0.001  NGI 1.01 (1.00-1.02),  p=0.066 | GCS 1.10 (1.06-1.14),  p<0.001  NGI 1.01 (1.00-1.02),  p=0.277 | LAS 1.20 (1.09-1.32),  p<0.001  NGI 1.01 (1.00-1.02),  p=0.165 |
| **Contractility** | | | | |
| Ees  0.71 (0.58-0.88),  p=0.002 | EF 0.94 (0.91-0.97),  p<0.001  Ees 1.01 (0.76-1.34),  p=0.956 | GLS 1.11 (1.06-1.17),  p<0.001  Ees 0.84 (0.68-1.03),  p=0.094 | GCS 1.10 (1.06-1.14),  p<0.001  Ees 0.85 (0.66-1.08),  p=0.175 | LAS 1.19 (1.09-1.31),  p<0.001  Ees 0.84 (0.67-1.05),  p=0.122 |
| **Ventriculo-arterial coupling** | | | | |
| Ea/Ees  1.83 (1.53-2.18),  p<0.001 | EF 0.97 (0.93-1.01),  p=0.095  Ea/Ees 1.42 (0.96-2.08),  p=0.076 | GLS 1.10 (1.04-1.15),  p<0.001  Ea/Ees 1.54 (1.23-1.92),  p<0.001 | GCS 1.08 (1.03-1.13),  p<0.001  Ea/Ees 1.44 (1.14-1.83),  p=0.003 | LAS 1.14 (1.03-1.25),  p=0.008  Ea/Ees 1.57 (1.24-2.00),  p<0.001 |
| Data is reported for patients ≥65 years of age (n=547, MACE n=55) and presented as hazard ratio with 95% confidence interval and p-value based on cox-regression analyses. Ea: effective arterial elastance, NGI: non-geometric left ventricular end-systolic afterload index, Ees: end-systolic elastance. | | | | |

| **Table S9** Interaction of LV functional and loading parameters regarding major adverse cardiac events  in STEMI | | | | |
| --- | --- | --- | --- | --- |
| **Variable/Univariate** | Multivariate  LVEF | Multivariate  GLS | Multivariate  GCS | Multivariate  LAS |
| **Afterload** | | | | |
| Ea  1.32 (1.17-1.49),  p<0.001 | EF 0.94 (0.91-0.96),  p<0.001  Ea 1.10 (0.95-1.28),  p=0.210 | GLS 1.13 (1.07-1.19),  p<0.001  Ea 1.17 (1.00-1.37),  p=0.058 | GCS 1.09 (1.04-1.13),  p<0.001  Ea 1.18 (1.04-1.35),  p=0.012 | LAS 1.25 (1.14-1.38),  p<0.001  Ea 1.20 (1.03-1.39),  p=0.018 |
| NGI  1.02 (1.01-1.03),  p<0.001 | EF 0.93 (0.90-0.96),  p<0.001  NGI 1.00 (0.98-1.01),  p=0.576 | GLS 1.13 (1.07-1.19),  p<0.001  NGI 1.01 (1.00-1.03),  p=0.031 | GCS 1.09 (1.04-1.14),  p<0.001  NGI 1.01 (1.00-1.02),  p=0.236 | LAS 1.25 (1.13-1.37),  p<0.001  NGI 1.01 (1.00-1.02),  p=0.089 |
| **Contractility** | | | | |
| Ees  0.66 (0.51-0.86),  p=0.002 | EF 0.92 (0.89-0.95),  p<0.001  Ees 1.19 (0.86-1.65),  p=0.283 | GLS 1.13 (1.07-1.19),  p<0.001  Ees 0.84 (0.65-1.07),  p=0.161 | GCS 1.09 (1.04-1.14),  p<0.001  Ees 0.87 (0.67-1.13),  p=0.283 | LAS 1.25 (1.14-1.38),  p<0.001  Ees 0.86 (0.67-1.11),  p=0.236 |
| **Ventriculo-arterial coupling** | | | | |
| Ea/Ees  1.97 (1.67-2.32),  p<0.001 | EF 0.97 (0.93-1.00),  p=0.057  Ea/Ees 1.54 (1.11-2.15),  p=0.010 | GLS 1.10 (1.04-1.16),  p=0.002  Ea/Ees 1.65 (1.33-2.05),  p<0.001 | GCS 1.06 (1.00-1.11),  p=0.033  Ea/Ees 1.65 (1.30-2.09),  p<0.001 | LAS 1.19 (1.08-1.31),  p<0.001  Ea/Ees 1.67 (1.34-2.08),  p<0.001 |
| Data is reported for STEMI patients (n=782, MACE n=53) and presented as hazard ratio with 95% confidence interval and p-value based on cox-regression analyses. Ea: effective arterial elastance, NGI: non-geometric left ventricular end-systolic afterload index, Ees: end-systolic elastance. | | | | |

| **Table S10** Interaction of LV functional and loading parameters regarding major adverse cardiac events  in NSTEMI | | | | |
| --- | --- | --- | --- | --- |
| **Variable/Univariate** | Multivariate  LVEF | Multivariate  GLS | Multivariate  GCS | Multivariate  LAS |
| **Afterload** | | | | |
| Ea  1.26 (0.94-1.68),  p=0.122 | EF 0.95 (0.92-0.99),  p=0.016  Ea 1.06 (0.78-1.44),  p=0.713 | GLS 1.13 (1.05-1.20),  p<0.001  Ea 1.12 (0.83-1.50),  p=0.462 | GCS 1.06 (1.00-1.12),  p=0.037  Ea 1.18 (0.86-1.61),  p=0.304 | LAS 1.23 (1.08-1.41),  p=0.002  Ea 1.08 (0.80-1.47),  p=0.611 |
| NGI  1.01 (1.00-1.03),  p=0.059 | EF 0.95 (0.91-0.99),  p=0.028  NGI 1.00 (0.98-1.02),  p=0.959 | GLS 1.12 (1.05-1.20),  p=0.001  NGI 1.01 (0.99-1.02),  p=0.374 | GCS 1.05 (0.99-1.11),  p=0.134  NGI 1.01 (1.00-1.03),  p=0.171 | LAS 1.23 (1.06-1.42),  p=0.005  NGI 1.00 (0.99-1.02),  p=0.678 |
| **Contractility** | | | | |
| Ees  0.84 (0.63-1.11),  p=0.211 | EF 0.94 (0.90-0.99),  p=0.011  Ees 1.12 (0.77-1.61),  p=0.560 | GLS 1.13 (1.05-1.20),  p<0.001  Ees 0.92 (0.69-1.22),  p=0.559 | GCS 1.05 (1.00-1.12),  p=0.069  Ees 0.87 (0.62-1.23),  p=0.424 | LAS 1.24 (1.08-1.42),  p=0.002  Ees 0.96 (0.72-1.28),  p=0.772 |
| **Ventriculo-arterial coupling** | | | | |
| Ea/Ees  1.96 (1.29-2.96),  p=0.001 | EF 0.97 (0.88-1.06),  p=0.454  Ea/Ees 1.28 (0.39-4.28),  p=0.684 | GLS 1.11 (1.03-1.20),  p=0.004  Ea/Ees 1.47 (0.92-2.35),  p=0.109 | GCS 1.03 (0.97-1.10),  p=0.366  Ea/Ees 1.85 (1.10-3.12),  p=0.021 | LAS 1.19 (1.02-1.39),  p=0.029  Ea/Ees 1.33 (0.76-2.32),  p=0.321 |
| Data is reported for NSTEMI patients (n=351, MACE n=25) and presented as hazard ratio with 95% confidence interval and p-value based on cox-regression analyses. Ea: effective arterial elastance, NGI: non-geometric left ventricular end-systolic afterload index, Ees: end-systolic elastance. | | | | |
